# Effect of Ag Addition on the Electrochemical Performance of Cu10Al in Artificial Saliva

**DOI:** 10.1155/2016/4792583

**Published:** 2016-08-29

**Authors:** R. J. Salgado-Salgado, J. Porcayo-Calderon, O. Sotelo-Mazon, R. A. Rodriguez-Diaz, G. Salinas-Solano, V. M. Salinas-Bravo, L. Martinez-Gomez

**Affiliations:** ^1^Facultad de Ciencias Químicas e Ingeniería, Universidad Autónoma del Estado de Morelos, Avenida Universidad 1001, 62209 Cuernavaca, MOR, Mexico; ^2^CIICAp, Universidad Autónoma del Estado de Morelos, Avenida Universidad 1001, 62209 Cuernavaca, MOR, Mexico; ^3^Instituto de Ciencias Físicas, Universidad Nacional Autónoma de México, Avenida Universidad s/n, 62210 Cuernavaca, MOR, Mexico; ^4^Universidad Politécnica del Estado de Morelos, Boulevard Cuauhnahuac 566, Col. Lomas del Texcal, 62574 Jiutepec, Morelos, Mexico; ^5^Instituto de Investigaciones Eléctricas, Avenida Reforma 113, Colonia Palmira, 62490 Cuernavaca, MOR, Mexico; ^6^Corrosion y Protección (CyP), Buffon 46, 11590 México City, DF, Mexico

## Abstract

In this work we proposed to evaluate the corrosion resistance of four different alloys by electrochemical techniques, a binary alloy Cu10Al, and three ternary alloys Cu10Al-*x*Ag (*x* = 5, 10, and 15 wt.%) to be used like biomaterials in dental application. Biomaterials proposed were tested in artificial saliva at 37°C for 48 h. In addition, pure metals Cu, Al, Ag, and Ti as reference materials were evaluated. In general the short time tests indicated that the Ag addition increases the corrosion resistance and reduces the extent of localized attack of the binary alloy. Moreover, tests for 48 hours showed that the Ag addition increases the stability of the passive layer, thereby reducing the corrosion rate of the binary alloy. SEM analysis showed that Cu10Al alloy was preferably corroded by grain boundaries, and the Ag addition modified the form of attack of the binary alloy. Cu-rich phases reacted with SCN^−^ anions forming a film of CuSCN, and the Ag-rich phase is prone to react with SCN^−^ anions forming AgSCN. Thus, binary and ternary alloys are susceptible to tarnish in the presence of thiocyanate ions.

## 1. Introduction

Dental materials are specially elaborated materials, designed for use in odontology, and are intended to be utilized in the oral cavity. Metals and alloys have many applications in odontology. For example, steel alloys are typically utilized for the elaboration of instruments and wires for orthodontics. Gold alloys and alloys containing chromium are used for the fabrication of crowns, inlays, and denture bases whilst dental amalgam, an alloy that contains mercury, is the most widely used dental filling material. However, the fundamental requirement of the dental material is that this kind of biomaterial and the human tissue coexist without unwanted effects on each other. This property or condition is known as biocompatibility which can be defined as the capability or ability of a material to perform with a suitable and appropriate host response in a specific application [1].

Particularly, the nonnoble metals or the so-called base metals have been developed in order to be used in instruments for cleaning teeth, preparing teeth for restorations, and placing restorations. Base metals contain no gold, platinum, or palladium. Base metals are utilized in crowns for single tooth restorations and as frameworks for applications in bridges, partial dentures, and retainers. These kinds of metals are also utilized as wires and brackets to move or reposition teeth. Besides, they are also applicable as dental implants. The elevated durability and high strength of certain nonnoble metals make them a suitable choice for many of these applications and uses [2]. Nowadays, the nonnoble metals and alloys have been replacing the noble alloys, because of its lower cost and improved mechanical properties.

Corrosion resistance is a very relevant property for dental alloys, in addition to other properties such as ductility, strength, and casting accuracy. Corrosion of dental alloys in the oral environment not only induces a deterioration of restoration but also involves release of ions that is related directly to their biocompatibility [1, 2]. The biological consequences of corrosion products cannot be disregarded as these can be swallowed or absorbed through the oral tissues after they are dissolved. The study and assessment of the metal ion release that occurs in the oral cavity as a consequence of the corrosion process taking place when the dental alloy is exposed to the biological media is of great relevance because of the toxic effects that can produce the metallic ions in human organism. The search for metallic substitutes of noble alloys has been performed for many decades; of particular importance when developing and selecting base metal alloys is the evaluation of their corrosion properties. Cu-Al alloys show a good electrochemical performance, and they can be a substitute for gold-rich alloys used in dental applications. These alloys show an excellent gold color matching, and they can maintain brilliance in the oral environment. In some previous researches [3], noble metals like Ag have been added to base metals in order to enhance the corrosion resistance of these nonnoble alloys. A particular example is Ag which has an excellent corrosion resistance in many aqueous solutions and good biocompatibility. Some dental amalgams alloyed with Ag have been successfully utilized as dental materials [2, 4–6]. Takahashi et al. and Kikuchi et al. [7, 8] reported that adding ≤20 wt.% Ag can enhance the strength and grindability of cast Ti-alloys while preserving high levels of elongation (≥19%) during tensile tests. Shim et al. [9] reported that Ti-Ag alloys with low Ag contents (≤5 wt.%) have superior corrosion resistance compared to pure Ti and predicted that Ti-Ag alloys with higher Ag contents are less sensitive to F ions. That is why it is expected that the addition of Ag into Cu10Al alloys improves their corrosion resistance when evaluated in artificial saliva. Furthermore, the use of functional materials that inhibit colonization of oral bacteria is important in the development of biomaterials for dental applications [10].

## 2. Experimental Procedure

### 2.1. Sample Preparation

Four different alloys were made from pure electrolytic Cu (>99.9%), Al (99.9% pure), and Ag (99.9% pure): a binary alloy Cu10Al (wt.%) and three ternary alloys (Cu10Al-*x*Ag) with different additions of Ag (5, 10, and 15 wt.%). Pure elements were acquired with a local supplier of the metallurgical industry. An electric furnace was used to melt pure metals (Cu, Al, and Ag) using graphite crucibles, and ingots of 200 g were obtained; preferentially the alloys were recast two times for homogenizing and reducing porosity. Once this was done, the ingots are allowed to cool into the furnace to room temperature (about 24 hours). In addition, pure metals (Cu, Al, Ag, and Ti) also were evaluated, Ti as comparison specimen. Subsequently, all specimens were cut in squares of 5.0 × 5.0 × 3.0 mm using a diamond tipped blade. For electrical connection, specimens were spot-welded to a Cu insulated wire and then mounted in thermosetting resin. Sample surfaces were metallographically polished; the grinding process began with 120 grit sandpaper down to 1200 grit. Once the metallographically polished was complete, samples were washed with distilled water then by ethanol in an ultrasonic bath for 10 minutes and employed as the working electrode (WE).

### 2.2. Corrosive Electrolytes

Natural saliva is the most important fluid in the oral environment. However, its unstable nature does not make it suitable for* in vitro* studies and therefore the artificial saliva is used. In this work the corrosion performance of materials was evaluated in the artificial saliva proposed by Duffó and Quezada-Castillo [11].  Table 1 shows the chemical composition of the artificial saliva used in the electrochemical tests. Corrosion tests were carried out at 37°C in deaerated conditions.

### 2.3. Electrochemical Tests

Electrochemical tests were carried out using an ACM Instruments zero-resistance ammeter (ZRA) coupled to a personal computer. A three-electrode cell was used, whereas reference electrode (RE) was a saturated calomel electrode (SCE) and the counter electrode (CE) a Pt wire. Corrosion resistance of materials was calculated by potentiodynamic polarization from −400 mV to 1500 mV with respect to open circuit potential (*E*_corr_) at a sweep rate of 1 mV/s. Prior to the test, coupons were left to stabilize during 20 minutes. Electrochemical parameters (current density, *I*_corr_, Tafel slopes, *E*_corr_) were calculated using the extrapolation Tafel method from ±250 mV around corrosion potential (*E*_corr_). Cyclic polarization tests were used to evaluate the pitting corrosion resistance of all materials. WE was stabilized at the free corrosion potential for one hour, and then a potential sweep (0.166 mV/s) was done in the anodic direction until the potential reached a predetermined value. At this point, the scan direction was reversed until the hysteresis loop closes. In order to assess the ability of the materials to develop a protective oxide layer, the open circuit potential (OCP) as a function of time was measured during 48 hours. Linear polarization resistance (LPR) was measured by polarizing the materials from −20 to 20 mV around *E*_corr_ value at a scanning rate of 1 mV/s. LPR measurements were made during 48 hours. In order to investigate the morphology and the elements distribution of the reaction products, the corroded specimens were analyzed by scanning electron microscopy (SEM). Analyses were carried out using an X-ray energy dispersive (EDS) analyzer and a DSM 960 Carl Zeiss scanning electron microscope.

## 3. Results and Discussion

### 3.1. Potentiodynamic Polarization Curves

**Figure 1:**
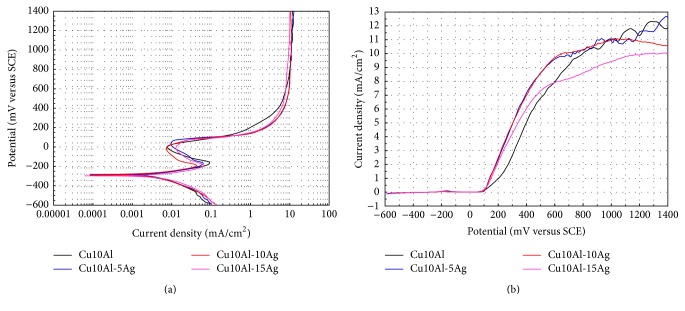
Polarization curves for the Cu10Al-*x*Ag (*x* = 0, 5, 10, and 15) alloys in artificial saliva at 37°C (d*E*/d*t* = 1.0 mV/s); (a) *E* versus log⁡*i*, (b) *i* versus *E*.

Potentiodynamic polarization curves for Cu10Al alloy and Cu10Al-*x*Ag ternary alloys are displayed in Figure 1 (due to the similarity of the curves, for clarity of the passive region the i-E plot is also included). The polarization curves for binary and ternary alloys exhibit an active-passive behavior. All alloys exhibit a wide passivation zone within the potential interval of −200 to 100 mV. It is noted that the Ag addition increases the range of the passive region of the base alloy. On the other hand, Ag addition induced a slight shift of the *E*_corr_ values of the base alloy towards more active values of potential. These results are consistent with other reported studies [3]. Also, Figure 1 shows that the corrosion rate expressed in terms of corrosion current density was diminished with the addition of Ag into the base alloy. So, the Ag addition induced a slight beneficial effect on the corrosion rate of the Cu10Al alloy. The corrosion rate values obtained are of the same order of magnitude as those reported for commercial CuAl based alloys [12]. The wide passivation zone observed in the polarization curves of the Cu-based alloys could be ascribed to the formation of a passive layer film probably composed by the Cu_2_O and CuO oxides. This statement is based in the results of previous research where the corrosion properties of palladium–silver–copper alloys exposed to artificial saliva were investigated and the presence of copper oxides in the corroded surface was revealed by the surface analysis conducted by the XPS technique [13].  Table 2 shows the electrochemical parameters for Cu10Al alloy and Cu10Al-*x*Ag ternary alloys.

**Figure 2:**
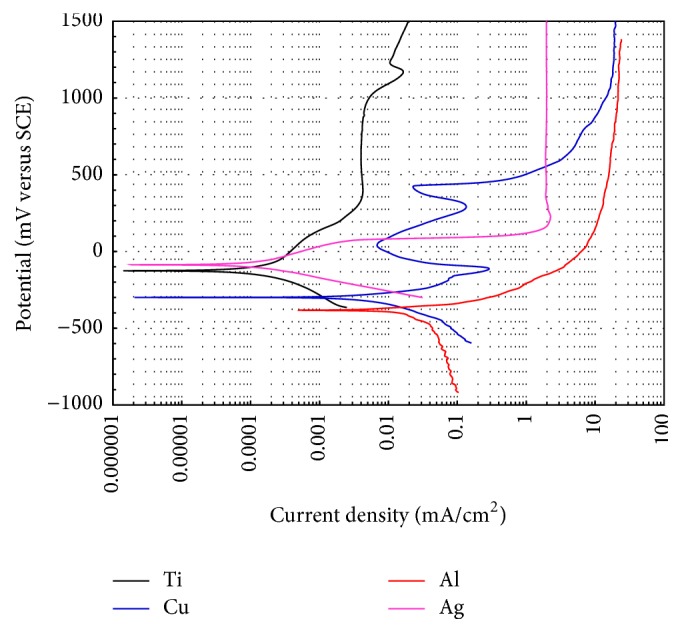
Polarization curves for the pure elements in artificial saliva at 37°C (d*E*/d*t* = 1.0 mV/s).

In order to understand the role of Al, Ag, and Cu on the corrosion behavior of the present CuAl based alloys, polarization curves of pure Al, Ag, and Cu elements are shown in Figure 2; also in this illustration is included the polarization curve of Ti element which is a typical metal used as dental implant and was included for comparison purposes. From this figure it can be observed that the noblest corrosion potential, *E*_corr_, corresponded to Ag, while Al element exhibited the most active corrosion potential with *E*_corr_ equal to −383 mV. The polarization curve of pure copper exhibited more defined passivation zones, showing in this case zones of primary and secondary passivation. Besides, pure Cu exhibited a nobler *E*_corr_ value than that of Al and also showed a minor corrosion rate. The passivation zones displayed by the polarization curves of pure Cu certainly are due to the formation of a corrosion product film composed by cuprous chloride, cuprous oxide, and cupric hydroxide, just as was stated in previous investigations [14] which studied the electrochemical corrosion behavior of Cu when exposed to chloride solution. In addition, pure Al displayed the highest corrosion rate of all pure elements and alloys with a corrosion current density of 0.38 mA/cm^2^. However, the Al addition into Cu does not reduce its rate of corrosion but, on the contrary, increases its corrosion resistance (Figure 1, Table 2). It is further noted that Al has no significant effect on the *I*/*E* response of copper; only at very anodic potential the relationship is affected by the disappearance of the second passive zone. Benedeti et al. [3] showed that the addition of Al produces a mixed surface layer (aluminium hydroxide-aluminium oxide-CuCl_ads_) which enhanced the corrosion resistance of pure copper. It is worth noticing that the *E*_corr_ values of Ti and Ag did not differ in a significant extent. This behavior could be related to the spontaneous oxidation of Ti to form a titanium oxide layer, despite that the standard reduction potentials of Ag and Ti differ in a great extent. The corrosion rate of Ti resulted to be less than that reported by Zhang et al. [15]. In their study, the authors reported *E*_corr_ and *I*_corr_ values for pure Ti of −68 mV and 0.0021 mA/cm^2^, respectively, and the passive film formed on surface of Ti was predominantly composed of TiO_2_, as determined by XPS. Polarization curve for pure Ag exhibited an activation polarization at low anodic overpotentials above *E*_corr_, followed by a limit current at potential of about 200 mV, while that Al reached also an anodic limit current near to 700 mV. Identical behavior has been reported in another work [16]. The activation polarization should be expected to be originated from the anodic dissolution of silver according to the following reaction:

(1) $$\begin{array}{c} Ag⟷{Ag}^{+}+e^{-} \end{array}$$

It is worth noticing that the corrosion rate of the Cu-Al alloy diminished after the Ag addition, where this behavior certainly is due to the modification of the corrosion product film formed on the Cu-Al alloy surface by AgCl and Ag_2_O. Furthermore, the Ag addition into binary alloy did not change appreciably the *I*/*E* relationship. Similar ternary alloys have been evaluated in acidic media and it has been found that the Ag addition does not appreciably modify the value of *E*_corr_; this is because the Ag does not undergo any transformation [17]. Similar results have been reported when CuAlAg alloys have been evaluated in rich electrolyte chlorides [3]. However, it is worth noting that the breaking point of the passive region of the ternary alloys is practically the same as that observed in Ag. The formation of these silver chloride and silver oxides was reported in a previous research [18], which studied the electrochemical corrosion behavior of pure Ag when exposed to Ringer's solution. Regarding the corrosion rate, the minor corrosion rate corresponded to Ag which showed almost the same value of that of Ti, while Al exhibited the greatest corrosion rate. This could be associated with the greater corrosion susceptibility of Al exposed to a chloride containing solution.  Table 2 shows the electrochemical parameters of the pure elements. It is important to recognize that the values shown in Table 2 are the electrochemical parameters corresponding to the beginning of corrosion process. However, these values may change as time passes. Basically this depends on the ability of materials to form a protective oxide on its surface (see sections of open circuit potential and linear polarization).

### 3.2. Cyclic Polarization Curves

Cyclic polarization measurements were performed to determine the susceptibility of materials to pitting corrosion; this is because any material or alloy for use as biomaterial should have good resistance to localized corrosion. In such curves, the area under the curve, of the hysteresis loop developed, indicates the extent of localized attack suffered by the material (current density during the reverse scan is higher than that for the forward scan). If a hysteresis loop is not formed, this indicates that the material has excellent resistance to localized attack (current density during the reverse scan is less than that for the forward scan). The interpretation of the hysteresis loop also provides two important potential values: the pitting potential (*E*_np_) and the protection potential (*E*_pp_). The first value indicates the potential at which pitting corrosion initiates and propagates (abrupt increase in the anodic current density at the point where the passive zone ends), and the second value indicates the potential at which localized attack ends, and the corrosion rate decreases significantly (potential at which the forward and the reverse scans intersect). Generally during the anodic polarization, formation and repair of the passive film are observed, and its rupture is clearly identified by the sudden increase in current density.

**Figure 3:**
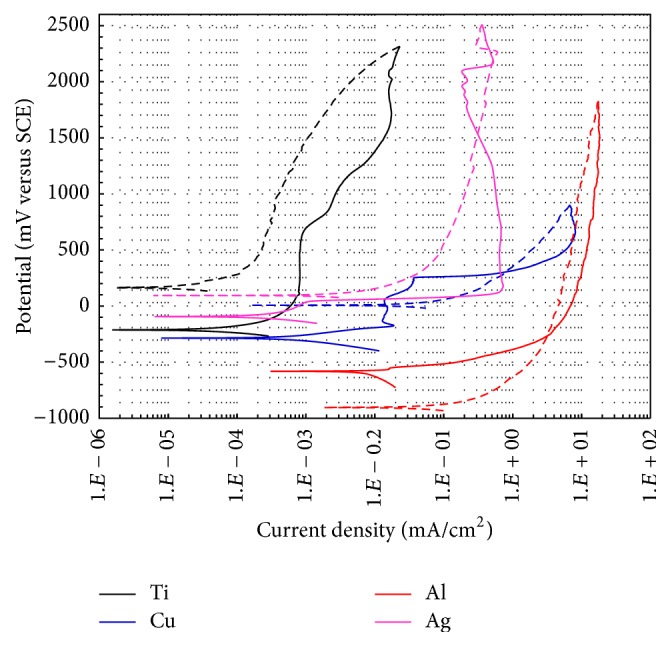
Cyclic polarization curves for pure elements (Ti, Cu, Al, and Ag) in artificial saliva at 37°C (d*E*/d*t* = 0.167 mV/s).

In Figure 3 is observed the anodic behavior of the pure elements from cyclic polarization curves. Ti shows a wide passive-pseudopassive zone without displaying a defined area of pitting potential, which is consistent with studies indicating the high resistance to pitting corrosion of titanium in chlorides-rich environments [19]. Cu exhibited a pitting potential of about 280 mV and a protection potential of −22 mV. However, Al showed a high susceptibility to pitting corrosion, because immediately above its *E*_corr_ (*E*_np_ = −565 mV) shows a large increase in its current density, and it develops a passive area, but at higher current densities; also it does not show a protective potential. On the other hand, both Ag and Al show a large increase in its current density without developing a passive zone (*E*_np_ = 30 mV); then they develop an extensive passive region, but without establishing a potential protection during the reverse sweep. It has been reported that the presence of SCN^−^ anions provokes pitting corrosion on the aluminum surface [20–22]. However, due to the high concentration of Cl^−^ ions, it is possible that the effect of SCN^−^ anions has not been relevant.

**Figure 4:**
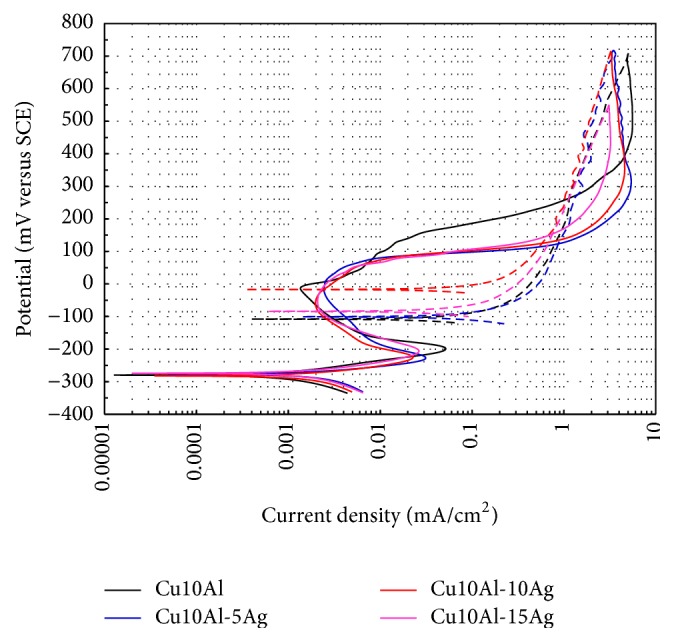
Cyclic polarization curves for the Cu10Al-*x*Ag (*x* = 0, 5, 10, and 15) alloys in artificial saliva at 37°C (d*E*/d*t* = 0.167 mV/s).

Figure 4 shows the anodic branch of the cyclic polarization curves of Cu10Al-*x*Ag alloys. It is observed that all Cu10Al-*x*Ag alloys exhibited an active-passive transition. In general, all alloys showed the same trend during the forward scan. However, it can be observed that the addition of Ag caused a sharp increase in the current density after pitting potential (*E*_np_). On the other hand, during the reverse scan it is observed that the addition of Ag (5 and 10 wt.%) increases the value of the protection potential (*E*_pp_), but higher Ag additions do not improve this value. From the area under the curve of the hysteresis loop developed it can be observed that the Ag addition reduces the extent of localized attack; the effect is greater with the addition of 10% Ag.

### 3.3. Open Circuit Potential Curves

**Figure 5:**
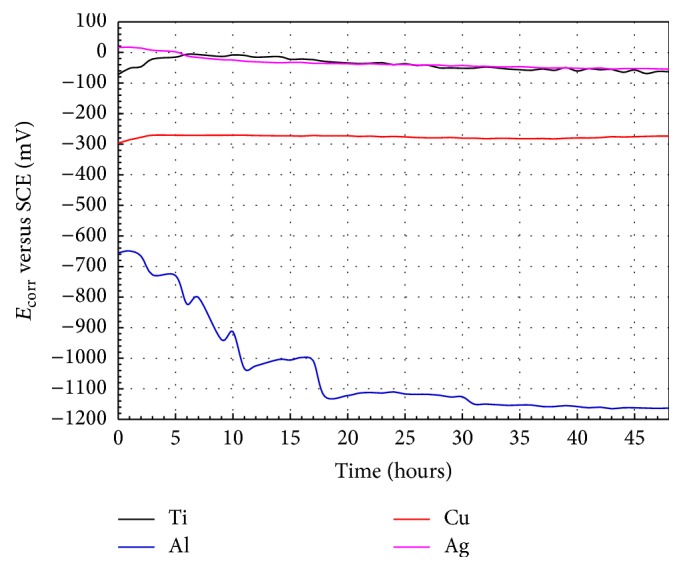
*E*_corr_ values after testing time for the pure elements in artificial saliva at 37°C.

The change of the *E*_corr_ values during an exposure time of 48 h for Ti, Al, Cu, and Ag in artificial saliva is showed in Figure 5. At the beginning of immersion, the *E*_corr_ values of Cu and Ti electrodes move towards nobler values. This behavior is probably due to the highest reactivity of Cu and Ti which induced an initial formation and growth of a passive oxide film. The initial increase of *E*_corr_ observed in Ti could certainly be ascribed to the formation of a layer composed by TiO_2_ [23–25]. Al showed a slight initial shift of *E*_corr_ toward less negative values, but after 1 h of immersion, corrosion potential exhibited a tendency to diminish in a great extent until a steady state was achieved at approximately 30 h of exposure. Besides, during the diminution of the corrosion potential values, oscillations in the active and noble directions were observed. This behavior is due to the initial formation of an Al-oxide passive film with a subsequent series of passivation-pitting-repassivation events; in this case the Cl^−^ ions contained in the artificial saliva originated the pitting corrosion process in the Al oxide passive film, where this kind of corrosion is typical of Al exposed to saline medium [26]. However, Ag showed a slight decrease in its corrosion potential values reaching values similar to those of Ti, after 20 hours of immersion. In all cases, after the initial change in the corrosion potential, a certain constant potential value (steady-state potential) was attained. Ti and Ag exhibited the nobler corrosion potentials as compared with those of Cu and Al during the 48 h of immersion. These findings are in agreement with the behavior displayed in polarization curves as shown in Figure 2 and Table 2. Similarly, the lowest *E*_corr_ corresponded to Al during the whole exposure time, which is also in accordance with the behavior displayed in polarization curves.

**Figure 6:**
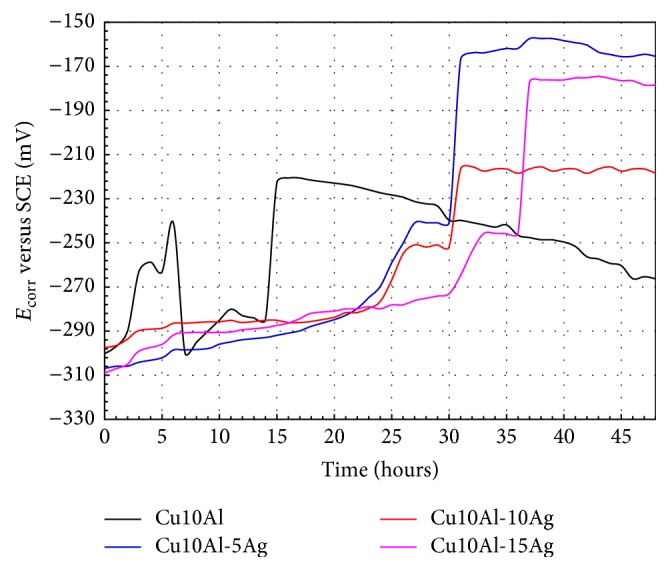
*E*_corr_ values after testing time for Cu10Al-*x*Ag alloys in artificial saliva at 37°C.

The change of the *E*_corr_ values with time for the Cu10Al alloys with different content of Ag is showed in Figure 6. From the first moments of immersion, the *E*_corr_ values of Cu10Al-*x*Ag alloys move towards to nobler values due to the initial formation and growth of a passive film. Alloy with Ag addition exhibited a more or less steady increment of *E*_corr_ while the exposure time had advanced up to 25–30 h of immersion; then, it can be deduced that the Ag addition originated a major stability of the passive film formed. In all cases, beyond 25–30 h immersion, an abrupt increase in the corrosion potential values was observed, subsequently reaching a steady state. This behavior can be certainly attributed to the formation of a stable passive film on the surface of the ternary alloys while the immersion time had elapsed. Binary Cu10Al alloy exhibited a less stable behavior. In this case, the *E*_corr_ values oscillated towards the noble and active directions, respectively, where this behavior is due to a successive series of passivation-pitting-repassivation processes of the passive film formed on surface of binary alloy. This in turn indicates that the passive layer was not stable, porous, or possessing little adherence with the metal alloy surface and could have been detached and also could be susceptible to pitting corrosion.

### 3.4. Linear Polarization Resistance Curves

**Figure 7:**
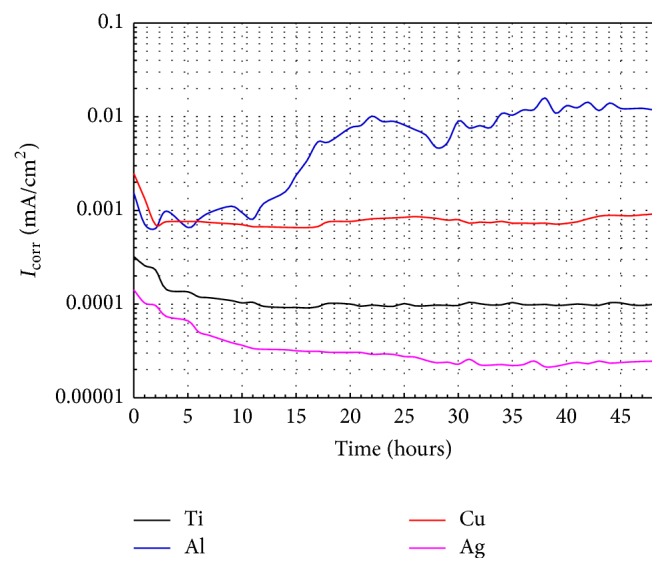
Change of *I*_corr_ values with time for the pure elements in artificial saliva at 37°C.

**Figure 8:**
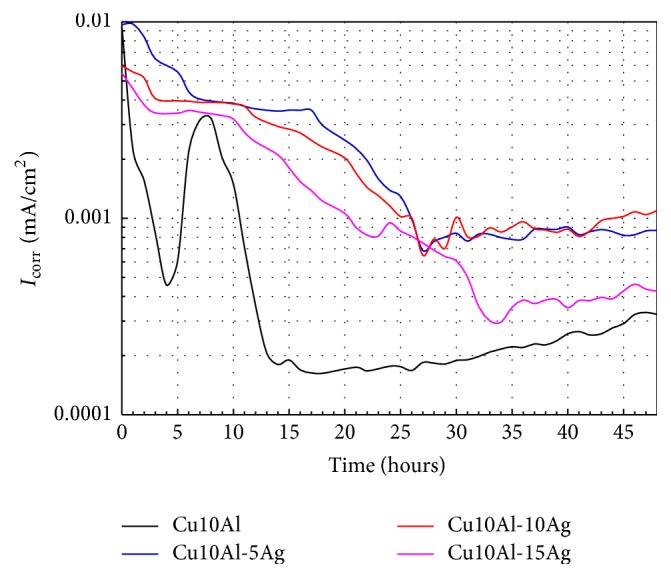
Change of *I*_corr_ values with time for the Cu10Al-*x*Ag alloys in artificial saliva at 37°C.

Progress of *I*_corr_ obtained by linear polarization measurements over time for all materials evaluated in artificial saliva solution is showed in Figures 7 and 8. Data was obtained from the polarization resistance measurements using Stern-Geary expression.

(2) $$\begin{array}{c} i_{corr}=\frac{b_{a}b_{c}}{2.303R_{p}\left(b_{a}+b_{c}\right)}, \end{array}$$

where the *b*_*a*_ and *b*_*c*_ values were those reported in Table 2.

Figure 7 displays the variation of *I*_corr_ as a function of exposure time for the pure elements (Al, Ti, Cu, and Ag). According to this illustration the lowest *I*_corr_ exhibited during the whole immersion time corresponded to Ag, and this behavior is logic because of the noble character of this element. Its corrosion rate tends to diminish as the exposure time had elapsed. This behavior is surely due to a passivation process of the Ag surface, due to the formation of AgCl and Ag_2_O as it has been reported previously [18]. Ti was the second element with the best performance, from 10 hours of immersion, Ti showed a constant corrosion rate, and this is related to the excellent chemical stability derived of the formation of a highly protective layer on its surface. In this case the formation of TiO_2_ oxide is responsible for the passivity of this element [25, 27]. Similarly, the corrosion rate of Cu decreased significantly during the first 3 hours of exposure and after that remained more or less constant until the 48 hours of immersion. This behavior is associated with the good stability of the passive film formed into copper surface. According to a previous research, this protective layer surely can be constituted by cuprite Cu_2_O, CuCl and/or Cu_2_Cl(OH_3_) [28], and/or CuS (as it will be observed in* SEM Analysis*). However, the variation of *I*_corr_ of pure Al exhibited an opposite trend, since the corrosion rate of pure Al increased in a fluctuating way during all the immersion time. This behavior is typical of Al when exposed to chloride media, because the passive film formed over the Al surface is frequently susceptible to localized breakdown producing in this way an accelerated dissolution of the underlying aluminum. In this case, if the attack initiates on an open surface, then the pitting corrosion process is produced [29].

Figure 8 displays the variation of *I*_corr_ as a function of exposure time for binary and ternary alloys. In this figure clearly is observed a decrease of the corrosion rate while the exposure time had elapsed, for all the tested alloys. This behavior is clearly associated with the good stability of the passive film formed onto alloys surface, especially the ternary ones. The behavior of the Cu10Al alloy shows both abrupt decreases and increases in its *I*_corr_ values in the first 13 hours of immersion. From this point the corrosion rate is the lowest of all tested alloys. However, its *I*_corr_ values show a steady increase in the rest of the test. On the other hand, the corrosion behavior of ternary alloys was different from that of binary alloy. Ternary alloys showed a similar trend, namely, a steady decrease in their corrosion rate values and then reaching stationary values. Cu10Al-5Ag and Cu10Al-10Ag had the same performance and Cu10Al-15Ag the best one. The different behavior of *I*_corr_ of binary alloy as compared with ternary alloys was originated by the addition of Ag, since this element induced a stabilization of the passive film formed on Cu10Al alloy while the immersion time had advanced. Based on previous reports [14, 28], the compounds formed on surface of Cu, Al, or Cu-Al alloys when they were exposed to the chloride solutions are typically Cu_2_O, Cu_2_Cl, Cu_2_Cl(OH_3_), or Al_2_O_3_. Therefore, the addition of Ag induced a modification of the copper oxide, copper chlorides, or aluminum oxide, which resulted in an enhancement of the passive character of the film.

### 3.5. SEM Analysis

**Figure 9:**
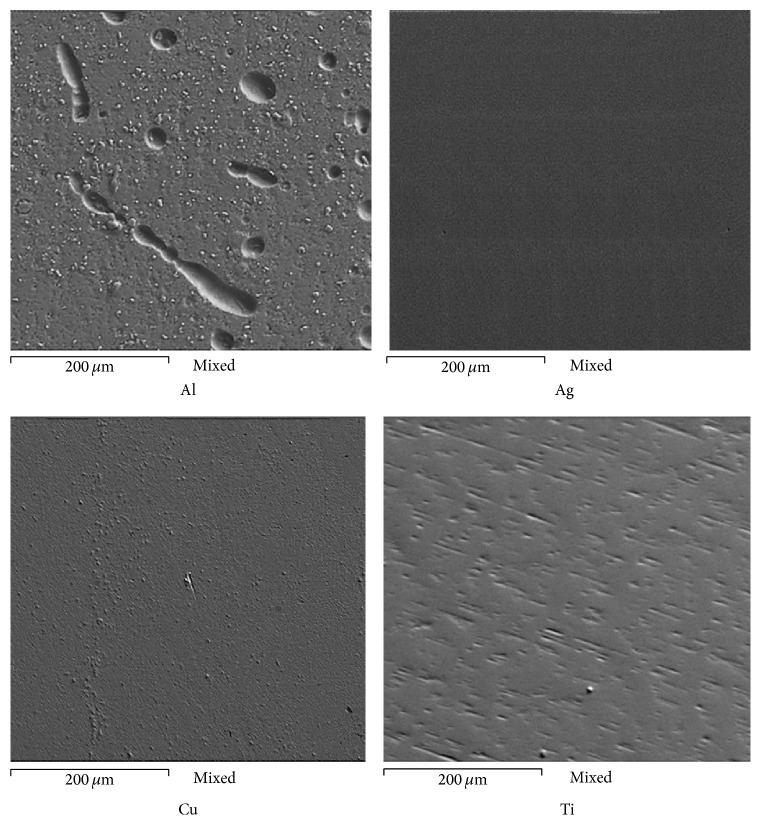
Superficial aspect for Al, Ag, Cu, and Ti, after the corrosion test in artificial saliva at 37°C for 48 hours.

Figure 9 shows the superficial appearance for Au, Ag, Cu, and Ti after evaluating its corrosion resistance in artificial saliva. It is noted that the Al underwent a process of localized corrosion, where some pits reached sizes greater than 200 microns. It is known that the corrosion behavior of Al depends on the specific activity of chloride ions present into electrolyte. Cl^−^ ions increase the anodic dissolution rate, besides causing pitting onto Al surface. Into the pits, there is a high concentration of both H^+^ ions and Cl^−^ ions, which stimulate the metal dissolution, besides the fact that the corrosion process is accelerated, and pitting attack is propagated because of the reduction of pH inside the pit [30]. These observations are consistent with what is previously discussed (electrochemical measurements), where this material showed the worst performance. It has been reported that the presence of SCN^−^ anions provokes pitting corrosion on the aluminum surface [20–22]. However, due to the high concentration of Cl^−^ ions, it is possible that the effect of SCN^−^ anions has not been relevant. By contrast, Cu showed a uniform corrosion process, and onto its surface a thin layer of corrosion products was observed, which according to EDS analysis is associated with the presence of sulfur. Joska et al. [13] reported that in environments containing thiocyanates Cu forms a CuSCN layer on its surface. Both Ti and Ag showed a surface free of deposits, but according to EDS analysis, in the case of Ag the presence of both oxygen and chlorine was detected; this can be associated with the formation of chlorides and oxides [3, 15, 16]; in the case of Ti only the presence of oxygen was detected, which may correspond to the formation of titanium oxide.

**Figure 10:**
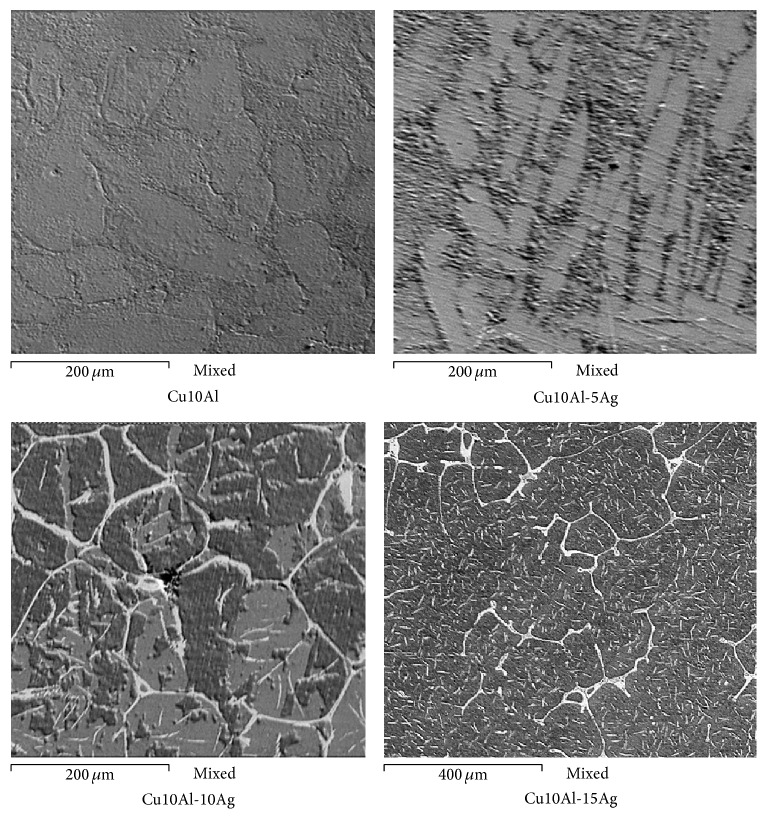
Superficial aspect of the Cu10Al-*x*Ag alloys after the corrosion test in artificial saliva at 37°C for 48 hours.

On the other hand, Figure 10 shows the superficial appearance for Cu10Al-*x*Ag alloys after evaluating its corrosion resistance in artificial saliva. In the case of Cu10Al alloy the accumulation of corrosion products is observed on its surface, being more noticeable in the grain boundaries. This may indicate that the Cu10Al alloy is preferably corroded by grain boundaries. This same behavior has been observed in other studies associating this type of attack with a dealuminification process of the grain boundaries [17]. According to EDS analysis, the corrosion products are mainly associated with sulfur, and since the accumulation of corrosion products was higher in the grain boundaries, then it is possible to say that the dealuminification process caused an increase in the concentration copper in this area, and therefore the corrosion rate observed was higher than that in the grain bulk. With the addition of 5 wt.% Ag, an area with a greater density of corrosion products was observed in areas that could correspond to grain boundaries, but without an apparent preferential attack in this area. EDS analysis indicated that the corrosion products are sulfur associated with Cu. It is clear that the addition of silver modified the form of attack of the alloy. A similar effect has been observed in other studies indicating that the addition of Silver enhances the corrosion resistance, due to the formation of AgCl and Ag_2_O [3, 15]. With an addition of 10 wt.% Ag, a surface with a higher density of corrosion products was observed. However, the density of corrosion products is virtually zero in Ag-enriched areas (grain boundaries). Similar morphological features were observed with the addition of 15 wt.% Ag. However, in this case it is possible to detect the presence of corrosion products in Ag-enriched areas. This agrees with the findings of Joska et al., who indicate that increasing the Ag content of the binary alloy is possible for the formation of a layer of AgSCN, but at low Ag concentrations longer exposure time is required [13].

**Figure 11:**
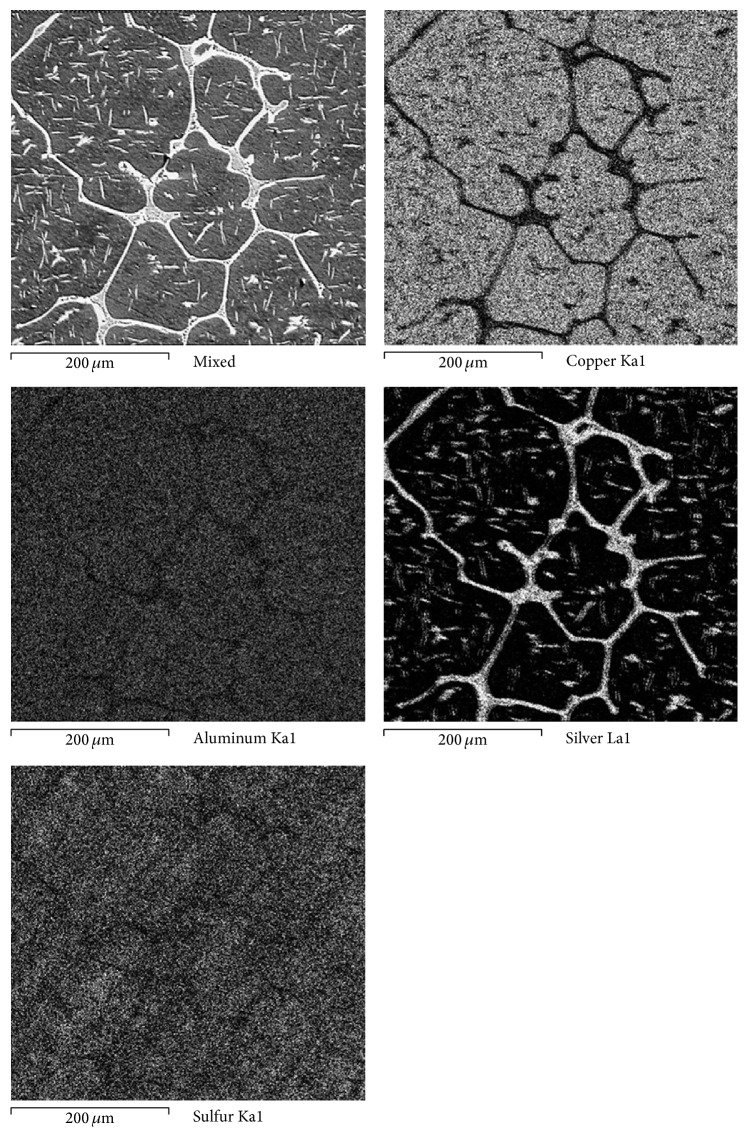
Superficial aspect of the Cu10Al-15Ag alloy and elements mapping after the corrosion test in artificial saliva at 37°C for 48 hours.

Figure 11 shows the morphology and the elements mapping of the Cu10Al-15Ag alloy at higher magnification; it can be seen that silver is segregated both in grain boundaries and intergranularly and that the sulfur concentration is greater in the bulk grain (rich in Cu and Al). It has been reported that in the Cu-Al-Ag alloy the present phases are similar to those present in the Cu-Al alloy [31], and the microstructure of the Cu-Al-Ag as-casting alloys is a Cu-Al-Ag solid solution (*α*_1_ phase) with the presence of a pearlitic phase (*γ*_1_ + *α*_1_, where *γ*_1_ = Cu_9_Al_4_) and Ag-rich precipitates in the grain boundaries [17]. The existence of different phases supports the formation of local cells, promoting the various possible redox reactions. According to experimental evidence, it is clear that the Ag-rich phase is the region where the cathodic reaction occurs mainly, and the anodic reaction occurs in *γ*_1_, *α*_1_ phases. Because of this as noted above, the *α*_1_ phase showed the greatest accumulation of corrosion products (mainly CuSCN) and the Ag-rich precipitates the lower density of corrosion products (mainly AgSCN).

The desired mechanical properties of an alloy for dental applications are Vickers hardness: 200–310, elastic modulus (GPa): 90–210, tensile strength (MPa): 580–1138, and specific gravity (g/cm^3^): 8–18 [32]. However, due to the wide range of these values, it is possible that most alloys meet the desired mechanical properties. On the other hand, the desirable values of corrosion resistance are uncertain, and simply it is wanted that the used alloys have a high corrosion resistance in order to prevent unwanted cytotoxic side-effects. This is particularly important when the alloy has a low content of nonnoble metals, or when the alloy is multiphase.

Degradation of dental materials is a complex mechanism where various physical and chemical processes act simultaneously (temperature, abrasion, attrition, fatigue, chemical degradation, etc.). From the viewpoint of chemical degradation, the primary condition of any metal to be used as biomaterial is that the corrosion products are not harmful to the body. The degradation of the tooth is essentially the dissolution of tooth mineral, and the external protective layer (enamel) is affected first [33]. Therefore, it can be said that the degradation of a biomaterial for dental applications must be equivalent to the* in vivo* wear experienced by the enamel. Some sources indicate that the* in vivo* enamel wear is about 100 microns/year [34].

For about 40 years, the Cu-Al based alloys are a substitute for conventional gold-rich alloys, due to a gold-like appearance and a more favorable price than alloys made from noble metals, and they are used for the economical fabrication of crowns and bridges [32, 35, 36]. However, due to the active nature of copper and aluminum, these alloys experience greater anodic dissolution and tarnishing. Thus, according to the experimental results shown, it can be said that the incorporation of Ag in the binary alloy (Cu10Al) contributes to reducing its anodic dissolution. However, these alloys are still susceptible to tarnishing due to the presence of thiocyanate ions (SCN^−^ anions), similar to that occurring in the presence of hydrogen sulfide and sulfide ions [37].

## 4. Conclusions

Corrosion tests were conducted to evaluate the effect of adding silver on the electrochemical performance of the binary alloy Cu10Al. The results showed that the Ag addition increases the range of the passive region of the base alloy, and also its corrosion rate was diminished. With the Ag addition the protection potential (*E*_pp_) was increased and the extent of localized attack was reduced. The best performance was associated with the stability of the passive film formed onto alloys surface. Cu10Al alloy is preferably corroded by grain boundaries, but with Ag addition, Ag-rich precipitates are formed in the grain boundaries, which prevent its corrosion. Binary and ternary alloys satisfy the requirements of a dental casting alloy. However, these alloys are prone to corrosion in presence of thiocyanate ions, Cu-rich phases form CuSCN, and in the alloys with high Ag content, the Ag-rich phases form AgSCN.

## Figures and Tables

**Table 1:** Chemical composition of artificial saliva (pH = 6.5) [11].

Compound	Content [g/L]
NaCl	0.600
KCl	0.720
CaCl_2_·2H_2_O	0.220
KH_2_PO_4_	0.680
Na_2_HPO_4_·12H_2_O	0.856
KSCN	0.060
NaHCO_3_	1.500
Citric acid	0.030

**Table 2:** Electrochemical parameters of the materials evaluated in artificial saliva at 37°C.

Material	*E*_corr_ (mV)	*B*_*a*_ (mV/Dec)	*B*_*c*_ (mV/Dec)	*I*_corr_ (mA/cm^2^)
Al	−383	94	1295	3.87*E* − 01
Cu	−299	115	204	1.04*E* − 02
Ag	−88	123	86	1.05*E* − 04
Ti	−125	287	172	1.48*E* − 04
Cu10Al	−285	72	183	4.23*E* − 03
Cu10Al-5Ag	−293	105	200	3.20*E* − 03
Cu10Al-10Ag	−288	92	180	2.60*E* − 03
Cu10Al-15Ag	−299	86	161	3.70*E* − 03
